# The History of the University of Maryland Dental Department

**Published:** 1889-11

**Authors:** 


### Editorial, Etc.

---

THE HISTORY OF THE UNIVERSITY OF MARYLAND DENTAL DEPARTMENT.—The University of Maryland, of which the Dental School forms one of the Departments, originally consisted of Medical, Law, Literary, and Theological Departments, the Medical School having been organized in the year 1807, by authority of a Charter granted by the General Assembly of the State of Maryland, and is the fourth in respect to date of organization, in the United States.

The Theological Department had a prosperous career until the establishment of purely Theological institutions, when it ceased to exist. The Literary Department also closed its doors on the death of the late Dr. Dalrymple, an eminent teacher and theologian.

The Medical and Law Departments, however, have had a continuous and prosperous existence since the date of their charters, and among their Alumni are to be found the names of many who have occupied some of the most exalted positions in their respective professions.

In the year 1837, and prior to the establishment of the first dental school in America, the first dental lectures to students were delivered in the University of Maryland by Dr. Chapin A. Harris and others in establishing the Baltimore College of Dental Surgery, the medical portion of the faculty of the said college being graduates of the Medical Department of the University of Maryland.

The University of Maryland Dental Department was organized in the year 1882, under a charter granted by the Legislature of Maryland at the session of 1881. The first regular session of the Dental Department was held during the winter of 1882 and '83, with sixty matriculates. The first graduating class consisted of thirty-four members.

Two years after the organization of the Dental Department, it was found necessary to enlarge the original dental building, (which was constructed for the practical teaching of dentistry, with unobstructed light on every side), in order to accomodate the increased number of students; and during the past summer another addition as large as the original building was erected, making an Infirmary about one hundred feet long by forty feet wide, with a corresponding increase in the size of the Laboratory which occupies the first floor, with the extracting, impression and reception rooms in the new wings.

Since its organization, there has been an annual increase in the number of students. The first session opened with sixty matriculates, and the first graduating class numbered thirty-four, five years or more of practice being then considered equivalent to one course of college instruction. The present class of the session of 1889 90 numbers between one hundred and thirty and one hundred and forty.

The government of the University of Maryland Dental Department is wholly vested in its faculty, all of its Professors being members of the Board of Regents of the entire university.

An extensive Museum Hall has also been added to the Dental Building to accomodate the large number of pathological and other specimens, collected from all parts of the world.

This school was the first to institute a Post-Graduate Course, although the credit of such an undertaking has been erroneously ascribed to another institution.

